# Quack Advertisements

**Published:** 1845-04

**Authors:** 


### QUACK ADVERTISEMENTS.

The English Evangelical Magazine, for January, contains the following announcement of the proprietors. Will the noble example ever be generally followed? If so, we may hope to see the end of quackery. Quacks live by the newspapers. It is their lying advertisements, stuffed with forged certificates, which give currency to their nostrums. The evil is a monstrous one, but we confess we have but little hope of living to see the day when the proprietors of newspapers shall with one accord set their faces against it.

"We shall cheerfully abandon, in future, the publication of all advertisements of quack medicines, which will be an act of homage to our own taste and judgment, no less than a concession to the strongly expressed opinions of some of our best friends, who, with ourselves, deeply deplore the disease and mortality occasioned by the nostrums of medical quacks, published daily in this great metropolis."

It is not too much to hope that the proprietors of religious journals may soon feel it their duty to take this stand. That would be a good deal gained. Many lives would be saved by it.

